# LncRNA-mediated ceRNA network reveals the mechanism of action of Saorilao-4 decoction against pulmonary fibrosis

**DOI:** 10.3389/fgene.2024.1339064

**Published:** 2024-03-12

**Authors:** Xinyue Fu, Xinni Song, Shufang Niu, Songli Shi, Hong Chang, Jun Qi, Peng Wang, Wanfu Bai

**Affiliations:** ^1^ Department of Pharmacy, Baotou Medical College, Baotou, China; ^2^ The First Affiliated Hospital of Baotou Medical College, Baotou, China; ^3^ The Second Affiliated Hospital of Baotou Medical College, Baotou, China

**Keywords:** Mongolian medicine, SRL-4 decoction, pulmonary fibrosis, lncRNA, ceRNA network

## Abstract

**Introduction:** Pulmonary fibrosis (PF), a type of interstitial pneumonia with complex etiology and high mortality, is characterized by progressive scarring of the alveolar interstitium and myofibroblastic lesions. In this study, we screened for potential biomarkers in PF and clarified the role of the lncRNA-miRNA-mRNA ceRNA network in the inhibitory effect of SRL-4 on PF.

**Methods:** Healthy male SPF SD rats were randomly divided into three groups, namely, CON, MOD, and SRL-4. Gene Ontology and Kyoto Encyclopedia of Genes and Genomes enrichment analyses were performed to determine the biological functions of the target genes. A visualized lncRNA-miRNA-mRNA ceRNA network was constructed using Cytoscape, while key genes in the network were identified using the cytoNCA plugin.

**Results:** Seventy-four differentially expressed lncRNAs and 118 differentially expressed mRNAs were identified. Gene Ontology analysis revealed that the target genes were mainly enriched in the cell membrane and in response to organic substances, while Kyoto Encyclopedia of Genes and Genomes analysis showed that the target genes were mainly enriched in the AMPK, PPAR, and cAMP signaling pathways. We elucidated a ceRNA axis, namely, Plcd3-OT1/rno-miR-150-3p/Fkbp5, with potential implications in PF. Key genes, such as AABR07051308.1-201, F2rl2-OT1, and LINC3337, may be important targets for the treatment of PF, while the AMPK, PPAR, and cAMP signaling pathways are potential key targets and important pathways through which SRL-4 mitigates PF.

**Conclusion:** Our findings suggest that SRL-4 improves PF by regulating the lncRNA-miRNA-mRNA network.

## 1 Introduction

Pulmonary fibrosis (PF) is a progressive and debilitating chronic interstitial lung disease with high incidence and mortality rates, limited treatment options, and extremely poor prognosis with a very low cure rate after diagnosis. In PF, damaged alveoli and lung cells are characterized by elevated inflammation, myofibroblast proliferation, and scar tissue formation (Hernandez et al., 2020). The exact etiology of PF remains unclear, and pathogenic factors or specific associations have not been determined. Prior to the development of PF, acute lung inflammation is often caused by viral infection, ionizing radiation, chemotherapy, air irritants, and pollutants (Fubini and Hubbard, 2003; Kligerman, 2022). Failure to resolve this inflammation in a timely manner can lead to the deposition of fibrotic tissue in the lungs and respiratory dysfunction (Noble et al., 2012). Many studies have clarified the complex pathophysiological mechanisms underlying PF, including genetics, epigenetics, microRNAs (miRNAs), cell signaling pathways, and apoptosis (Moss et al., 2022). Epigenetic modification such as DNA methylation, and regulation by miRNAs and lncRNA can affect the proliferation and maturation of epithelial cells. In 2014, pirfenidone and nintedanib were approved for PF treatment by FDA (Finnerty et al., 2021); however, their mechanisms of action remain poorly understood and side their effects remain challenging for patients. Currently, available treatments for PF can only slow the progression of the disease and cannot completely cure it; therefore, there remains an urgent need to elucidate the molecular mechanisms underlying PF, identify new diagnostic biomarkers and therapeutic targets, and develop effective treatments.

Long noncoding RNAs (lncRNAs) are a class of long-chain noncoding RNA molecules (>200 nucleotides) and important components of noncoding genes (Qian et al., 2019). LncRNAs regulate gene expression in different ways and interact with DNA, RNA, or proteins (Statello et al., 2021). LncRNAs can bind to specific sites on the miRNA, thereby buffering and reducing the ability of miRNAs to interfere with the mRNA-encoding proteins of target genes. This type of lncRNA is called a competitive endogenous RNA (ceRNA) (Jin et al., 2020). ceRNA-based regulatory mechanisms have been identified in various fibrotic diseases. For example, Yang et al. (2020) found that the lncRNA ZFAS5 acts as a ceRNA and sponges miR-38-1p to downregulate SLC1A150 expression, thereby attenuating ferroptosis and PF progression. In addition, lncRNA GAS5 upregulates ADAMTS-1 expression by endogenously adsorbing miR-21, promoting the degradation of ColI and ColIII and alleviating PF (Liu et al., 2020). However, our understanding of the ceRNA regulatory network in PF is limited, and further studies are warranted to reveal the unknown functions and mechanisms of the relevant ceRNAs.

Mongolian medicine Saorilao-4 decoction (SRL-4), also known as “Beisha Shen Siwei Tang,” comprises four herbs, namely, *Glehnia littoralis* Fr. Schmidt ex Miq. *Bistorta officinalis* Raf. *Glycyrrhiza uralensis* Fisch. and *Lithospermum erythrorhizon* Sieb. et Zucc. It is primarily used for clearing heat, resolving phlegm, and stopping cough (Tai and Lotus, 2022). Previously, we have reported that SRL-4 can significantly improve the progression of PF (Bai et al., 2021); however, the specific mechanism of action remains unclear. Therefore, in this study, we aimed to evaluate the effects of SRL-4 on PF and explores its potential molecular and signaling mechanisms. This study provides novel insights into the pharmacological potential and mechanism of action of SRL-4 in the treatment of PF.

## 2 Materials and methods

### 2.1 Experimental animal and grouping

Twenty-four healthy adult male Sprague–Dawley rats with body weights of 180–250 g were provided by the Chinese Academy of Food and Drug Testing [production license number: SCXK (Jing) 2017-0005] and were randomly divided into normal control (CON), model (MOD), and SRL-4 treatment groups, eight in each group. This work was approved by Baotou Medical College’s Animal Experimental Ethics Committee [No. (96) of 2022]. Except for the rats in the CON group, all rats were slowly injected with 5 mg/kg bleomycin at a concentration of 6 mg/mL into the trachea to induce PF. If the area of lung tissue damage exceeded 50%, the PF model was considered successful. SRL-4 (Batch number 20200414, specification 3 g per bag, with a mass ratio of 5:3:3:3 of Radix Glehniae-Laccifer locca Kerr-Licorice-Bistortae Rhizoma) purchased from the National Mongolian Medicine Formulation Center of Inner Mongolia International Mongolian Medicine Hospital. The preparation method is as follows: 3 g of herbal powder was added to 60 mL of 0.9% saline. The temperature of the solution was adjusted to approximately 40°C with stirring for 30 min until the powder was completely dissolved. Beginning from the first day after modeling, normal control group and model group rats were orally administered 10 mL/kg of physiological saline, while rats in each drug group were orally administered the corresponding dose of drug solution (dissolved in physiological saline), once a day, with an administration volume of 10 mL/kg, continuously for 4 weeks.

### 2.2 Determination of inflammatory cytokines and fibrosis-related indicators in rat lung tissues and observation of pathological changes in lung tissue of rats

An appropriate amount of right lung tissue was taken from rats in each group, and the levels of inflammatory cytokines, namely, interleukin-1 beta (IL-1β) and interleukin-6 (IL-6), as well as fibrosis-related indicators, namely, hyaluronidase (HA), laminin (LN), type III procollagen (PC-Ⅲ), and type IV collagen (Col-Ⅳ), in lung tissue were determined using an enzyme-linked immunosorbent assay kit and a microplate reader, according to the manufacturer’s recommendations.

Lung tissues from each group were collected for histopathological examination; tissue sections were then subjected to HE and Masson’s staining. The stained sections were examined using a light microscope to determine the pathological changes and collagen accumulation in the lung tissues, respectively.

### 2.3 Transcriptome sequencing

#### 2.3.1 Isolation, identification, and quantification of RNA

To perform RNA identification and quantification, we used the following methods: first, we used 1% agarose gel electrophoresis to identify RNA samples for possible contamination and degradation. Second, we used a NanoPhotometer to determine the purity and concentration of RNA with a NanoPhotometer^®^ spectrophotometer. Next, we used the RNA Nano 6000 assay to determine the integrity and quantity of RNA. Finally, we use the Agilent Bioanalyzer 2100 system’s RNA Nano 6000 assay kit for measurement. The combination of these methods ensures accurate identification and quantification of RNA, providing a reliable basis for subsequent RNA analysis.

#### 2.3.2 Library preparation for lncRNA and sequencing

The main factors for evaluating the samples are the RNA integrity (RIN value) and the total amount, which are the indicators for whether the library can be constructed normally. The starting amount for lncRNA library construction is 500 ng. The RNA area ranged from 61.6 to 189.7, the RNA concentration ranged from 90 to 545 ng/μL, and the minimum RIN score was 9.10 (See for Supplementary File S2 details). rRNA removal and strand-specific methods were used to prepare RNA libraries for lncRNA-seq. We used an rRNA Removal Kit to remove ribosomal RNA from total RNA, according to the manufacturer’s instructions, then RNA was fragmented into 250–300 bp and reverse transcription of the first-strand cDNA using dNTPs was performed. Next, we degraded RNA using 2 μL RNase H and synthesized second-strand cDNA using DNA polymerase I and dNTPs. The residual prominent ends of the double-stranded cDNA were converted to flat ends using exonuclease/polymerase activity. After adenylation of the 3′ end of the DNA fragment, the sequencing aptamer was ligated to the cDNA. To select 250–300-bp-long cDNA fragments, library fragments were purified using an AMPure XP system. Subsequently, uridylate digestion was performed using uridylate N-glycosylase, followed by PCR amplification to increase the number of cDNAs fragments.

After library construction was completed, a Qubit^®^ fluorometer was used to measure the concentration of the library and adjusted to 1 ng/μL. An Agilent 2100 Bioanalyzer was used to determine the insert sizes of the obtained libraries. Finally, qPCR was performed to accurately measure the concentration of cDNA library. Samples were sent for sequencing when the insert size and concentration of the library were consistent.

#### 2.3.3 Library preparation for small RNA and sequencing

A total amount of 2 μg total RNA per sample was used as input material for the small RNA library. Sequencing libraries were generated using NEBNext^®^ Multiplex Small RNA Library Prep Set for Illumina^®^ (NEB, United States) following manufacturer’s recommendations and index codes were added to attribute sequences to each sample. Briefly, NEB 3′ SR Adaptor was directly, and specifically ligated to 3′ end of miRNA, siRNA and piRNA. After the 3′ ligation reaction, the SR RT Primer hybridized to the excess of 3′ SR Adaptor (that remained free after the 3′ ligation reaction) and transformed the single-stranded DNA adaptor into a double-stranded DNA molecule. This step is important to prevent adaptor-dimer formation, besides, dsDNAs are not substrates for ligation mediated by T4 RNA Ligase 1 and therefore do not ligate to the 5′ SR Adaptor in the subsequent ligation step. 5′ends adapter was ligated to 5′ends of miRNAs, siRNA and piRNA. Then first strand cDNA was synthesized using 2 μL M-MuLV Reverse Transcriptase (RNase H–). PCR amplification was performed using LongAmp Taq 2X Master Mix, SR Primer for illumina and index (X) primer. PCR products were purified on a 8% polyacrylamide gel (100 V, 80 min). DNA fragments corresponding to 140–160 bp (the length of small noncoding RNA plus the 3′ and 5′ adaptors) were recovered and dissolved in 8 μL elution buffer. At last, library quality was assessed on the Agilent Bioanalyzer 2100 system using DNA High Sensitivity Chips.

#### 2.3.4 Quality control, mapping, and assembly of raw data

We processed the raw data (raw reads) using the FASTP (version 0.19.7) software. In this step, we obtained clean data (clean reads) by removing the following reads: 1) reads with 5′ adapters; 2) reads without 3′ adapters or insertion sequences; 3) reads with more than 10% of N bases; 4) reads with more than 50% of bases with quality values lower than Qphred ≤20 reads; 5) reads with multiple A/T/G/C. All downstream analyses were performed using clean, high-quality data. Clean reads from each sample were first mapped to the reference genome using the HISAT2 software. Reads alignment results were transferred to the program StringTie for transcript assembly.

### 2.4 Transcriptome data analysis

#### 2.4.1 Identification, quantification, and differential expression analysis of lncRNAs

We used Cuffmerge software to combine all transcripts. In this step, we obtained clean data (clean reads) by removing the following reads: 1) reads with 5′ adapters; 2) reads without 3′ adapters or insertion sequences; 3) reads with more than 10% of N bases; 4) reads with more than 50% of bases with quality values lower than Qphred ≤20 reads; 5) reads with multiple A/T/G/C. The new lncRNAs were named using the HUGO Gene Nomenclature Committee rules. We compared the new lncRNA features with those of known lncRNAs and mRNAs to confirm that they were lncRNAs.

EdgeR (3.22.5) was used for differential expression analysis. The resulting *p*-values were adjusted using the Benjamini–Hochberg method to control for false discovery rates. We defined the results as the differential expression of genes with |log2 (fold change) |>1 and padj<0.05.

#### 2.4.2 Identification, quantification, differential expression, and target gene prediction of miRNA

RNA degradation and contamination was monitored on 1% agarose gels. RNA purity was checked using the NanoPhotometer^®^ spectrophotometer (IMPLEN, CA, United States). RNA concentration was measured using Qubit^®^ RNA Assay Kit in Qubit^®^ 2.0 Flurometer (Life Technologies, CA, United States). RNA integrity was assessed using the RNA Nano 6000 Assay Kit of the Agilent Bioanalyzer 2100 system (Agilent Technologies, CA, United States).

MiRNA expression levels were estimated using the transcript per million (TPM) value as per the following criteria: normalization formula: normalized expression = mapped read count/Total reads*1,000,000.

For the samples with biological replicates, differential expression analysis of two conditions/groups was performed using the DESeq R package (1.8.3). The *p*-values was adjusted using the Benjamini- Hochberg method. Corrected *p*-value of 0.05 was set as the threshold for significantly differential expression by default.

For the samples without biological replicates, differential expression analysis of two samples was performed using the DEGseq (2010) R package. *p*-value was adjusted using q-value. Q-value<0.01 and |log2 (foldchange)|>1 was set as the threshold for significantly differential expression by default.

Miranda was used to predict the target genes of miRNA.

#### 2.4.3 LncRNA target gene prediction and Gene Ontology (GO) and Kyoto Encyclopedia of Genes and Genomes (KEGG) enrichment analysis

Two methods were used to predict the target genes of the lncRNAs, namely, cis-acting target gene prediction and trans-acting target gene prediction. Based on the theory of cis-regulatory elements, we selected protein-coding genes within 10/100 kb of lncRNAs as potential cis-acting targets. To predict trans-acting targets, we calculated Pearson’s correlation coefficients between coding genes and lncRNAs and identified the trans-acting regulatory elements. Trans-acting regulatory elements were identified in more than five samples to ensure an adequate sample size.

In living organisms, different genes function together to perform various biological functions. Pathway enrichment analysis can be used to explore the major biochemical, metabolic, and signaling pathways in which differentially expressed genes are involved, thus gaining a deeper understanding of the important roles of these genes in biological processes. The clusterProfiler R package was used for GO and KEGG enrichment analysis of target genes of DElncRNAs to further understand the role of lncRNAs and their target genes in biological functions and regulatory mechanisms. Gene length bias was also corrected, and enrichment was considered significant when the corrected *p*-value was <0.05.

### 2.5 Construction of ceRNA networks

LncRNA can adsorb miRNA by binding with it, and has the function of miRNA sponge. Using the principle of miRNA interference or inhibition of target genes, lncRNA targeted by miRNA and mRNA targeted by miRNA can be predicted. The correlation coefficient between miRNA and lncRNA is calculated, and select the results with a correlation coefficient threshold of *p* <−0.85 as the final result; the correlation coefficient between miRNA and mRNA is calculated, and select the results with a correlation coefficient threshold of *p* <−0.85 as the final result. Based on the research results of ceRNA, mRNA and lncRNA co-regulated by miRNA are selected. Utilizing the ceRNA hypothesis, we constructed an lncRNA-miRNA-mRNA network as follows: 1) Extract the miRNA-mRNA interaction information from miRanda and TargetScan, as well as the miRNA-lncRNA interaction information from miRanda; 2) If both lncRNA and mRNA are targeted and co-expressed negatively with a common miRNA, then the lncRNA-miRNA-mRNA trio is identified as a co-expression competing triplet, and the corresponding ceRNA regulatory network is constructed. Finally, Cytoscape software (version 3.7.1) was used to visualize the results, and the CytoNCA plugin in Cytoscape was used to identify the key genes in the network. Finally, Cytoscape software (version 3.7.1) was used to visualize the results, and the CytoNCA plugin in Cytoscape was used to identify the key genes in the network.

## 3 Results

### 3.1 Changes in the levels of inflammatory cytokines and fibrosis-related markers in rat lung tissue

**FIGURE 1:**
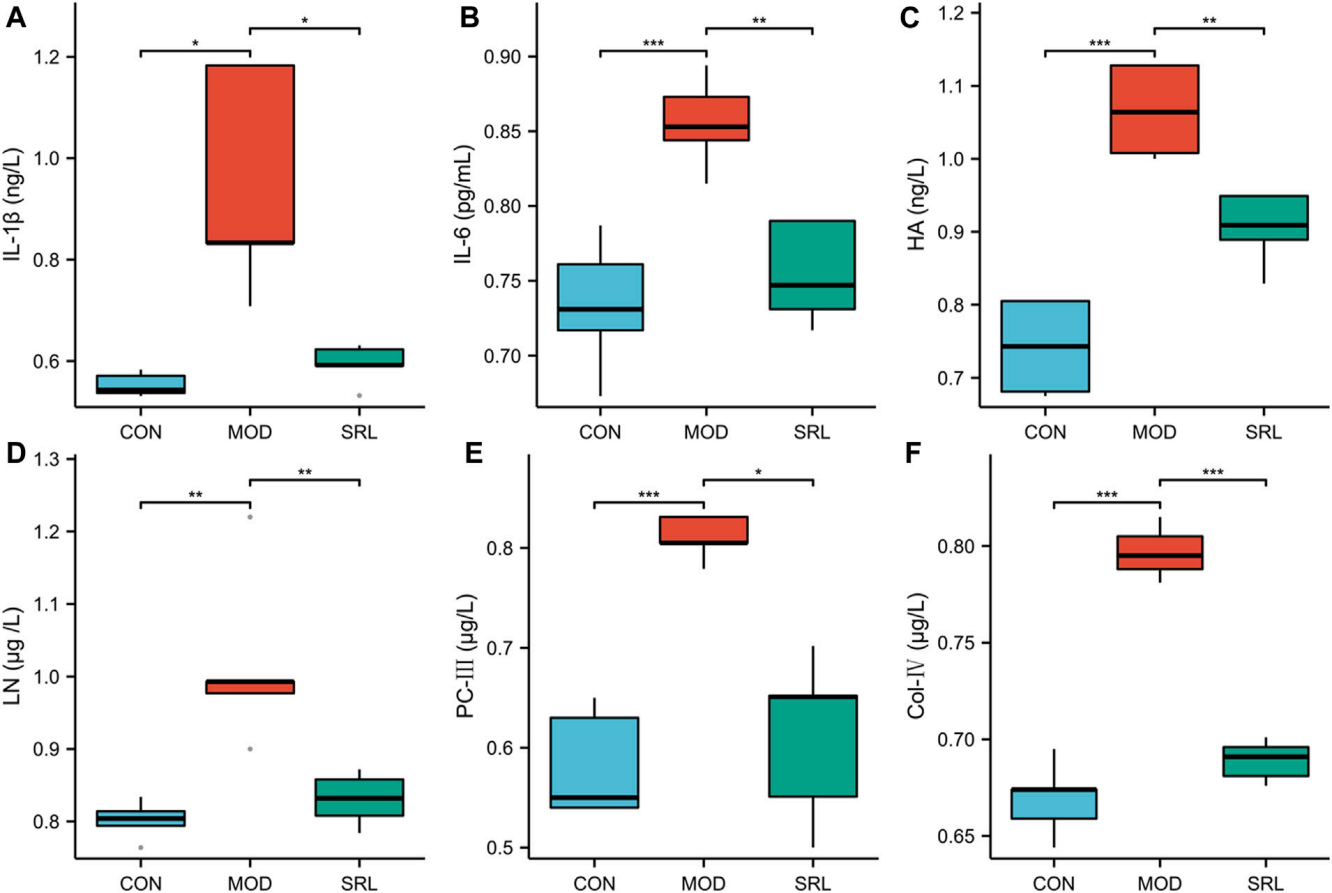
Changes in the levels of inflammatory factors and fibrosis-related indicators in the CON, MOD and SRL-4 groups. **(A)** IL-1β; **(B)** IL-6; **(C)** HA; **(D)** LN; **(E)** PC-III; **(F)** Col-IV.

Compared with those in the CON group, the levels of IL-1β, IL-6, HA, LN, PC-Ⅲ, and Col-IV were significantly increased in the lung tissues of rats in the MOD group (*p* < 0.01); compared with those in the MOD group, the levels of IL-1β, IL-6, HA, LN, PC-III, and Col-IV in the lung tissues of rats in the SRL-4 were significantly lower (*p* < 0.01) (Figure 1). Notably, there was a slight increase in inflammatory cytokines and fibrosis-related markers in the lung tissue of rats in the SRL-4 group compared to the CON group, but the overall indicators decreased significantly and were close to those of the CON group. The administration of SRL-4 drug has had a positive impact on the inflammatory and fibrotic processes, potentially through inhibiting inflammatory signaling pathways, or influencing the expression of fibrosis-related genes. However, further validation and research are required to confirm these findings.

### 3.2 Pathological changes in lung tissue of rats

**FIGURE 2:**
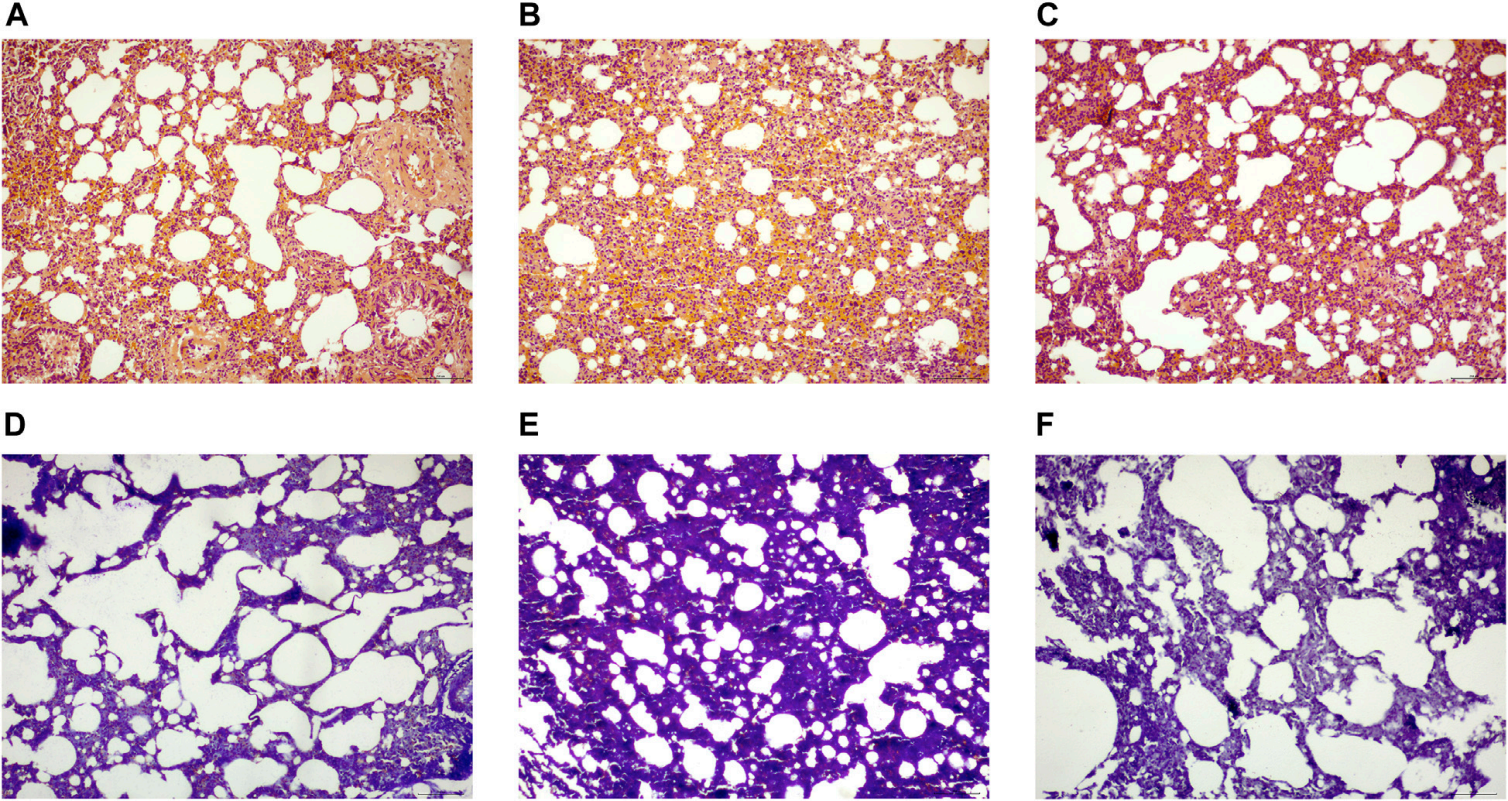
Histopathological changes of lung tissue in each group [H&E staining **(A–C)**, (×200)] and Masson staining **(D–F)**, (×200). **(A,D)**: normal group. **(B,E)**: model group. **(C,F)**: SRL-4 group.

The HE staining revealed that the alveolar structure of the rats in the CON remained intact, with normal alveolar wall epithelial cell morphology, normal alveolar septum without thickening, and no appreciable inflammatory cell infiltration in the interstitium. The alveolar structure of the rats in the MOD group was destroyed, some alveolar walls were fused and collapsed, the interstitium was hyperplastic and heavily infiltrated by inflammatory cells, the number of alveolar wall epithelial cells was significantly reduced, and the morphology of these cells was damaged and irregular. Compared to those in the MOD group, alveolar structure, alveolar wall epithelial cell morphology and number, and the degree of interstitial inflammation in the pulmonary interstitium were all significantly better in the SRL-4 group (Figures 2A–C). Masson’s staining revealed the alveolar structure of the rats in CON group, the alveolar septa was normal, and the absence of apparent deposition of blue-purple collagen fibers in the interstitium of the pulmonary mass. The alveolar structure of the rats in the mOD group was damaged, the alveolar septum was noticeably thickened, and there were significant deposits of lamellar blue-purple collagen fibers in the interstitium when compared to the normal group. The alveolar structure, alveolar septum, interstitial inflammation, and collagen fiber deposition were all significantly improved in the SRL-4 rats compared to those in the MOD group (Figures 2D–F).

### 3.3 Principal component analysis (PCA) and sample correlation analysis

**FIGURE 3:**
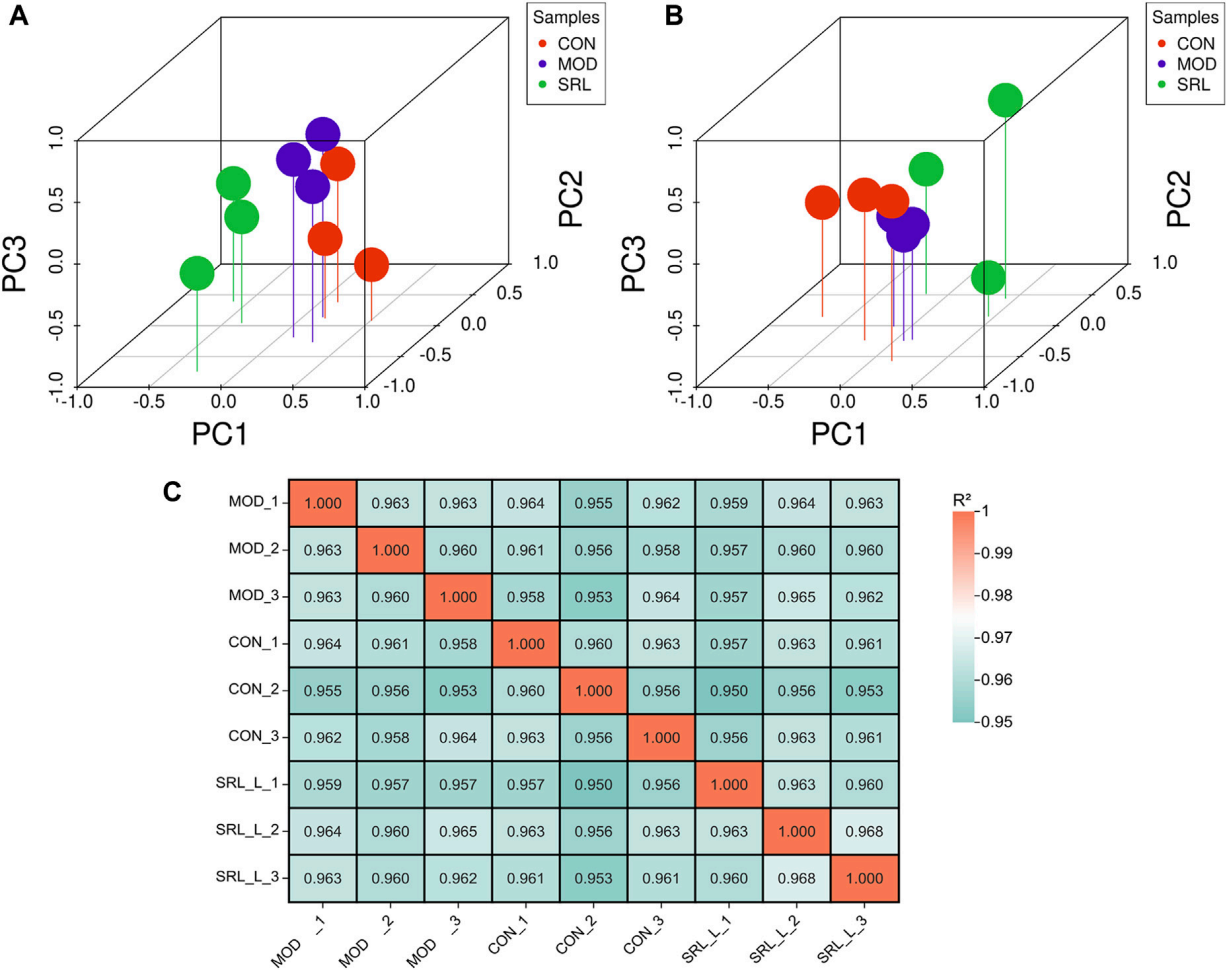
Principal component analysis (PCA) of DElncRNA and DEmRNA. **(A)** PCA (3D) of DElncRNA. **(B)** PCA (3D) of DEmRNA. **(C)** Heat map of sample correlation analysis.

Based on the differentially expressed lncRNAs in the CON, MOD, and SRL-4 groups, the distribution characteristics of the principal components of each group were explored using PCA. In a three-dimensional space, all the data points were distributed around the coordinate axes, and the distribution was relatively dispersed, indicating differences between the data points. In DElncRNA, the PC2 of SRL-4 has a higher dispersion trend compared to MOD (Figure 3A). In DEmRNA, the PC3 of SRL-4 has a higher dispersion trend compared to MOD (Figure 3B). We performed the same analysis on mRNAs and found that the differences among the three groups were smaller than those for lncRNAs. A correlation analysis was performed for each sample in the CON, MOD, and SRL-4 groups for subsequent differential gene analysis (Figure 3C). High Pearson correlation coefficients within each group indicated good repeatability; the higher the correlation coefficient between the samples within the group, the more reliable the differential genes, which could be used for subsequent differential gene analysis.

### 3.4 Identification of differentially expressed lncRNAs and mRNAs

**FIGURE 4:**
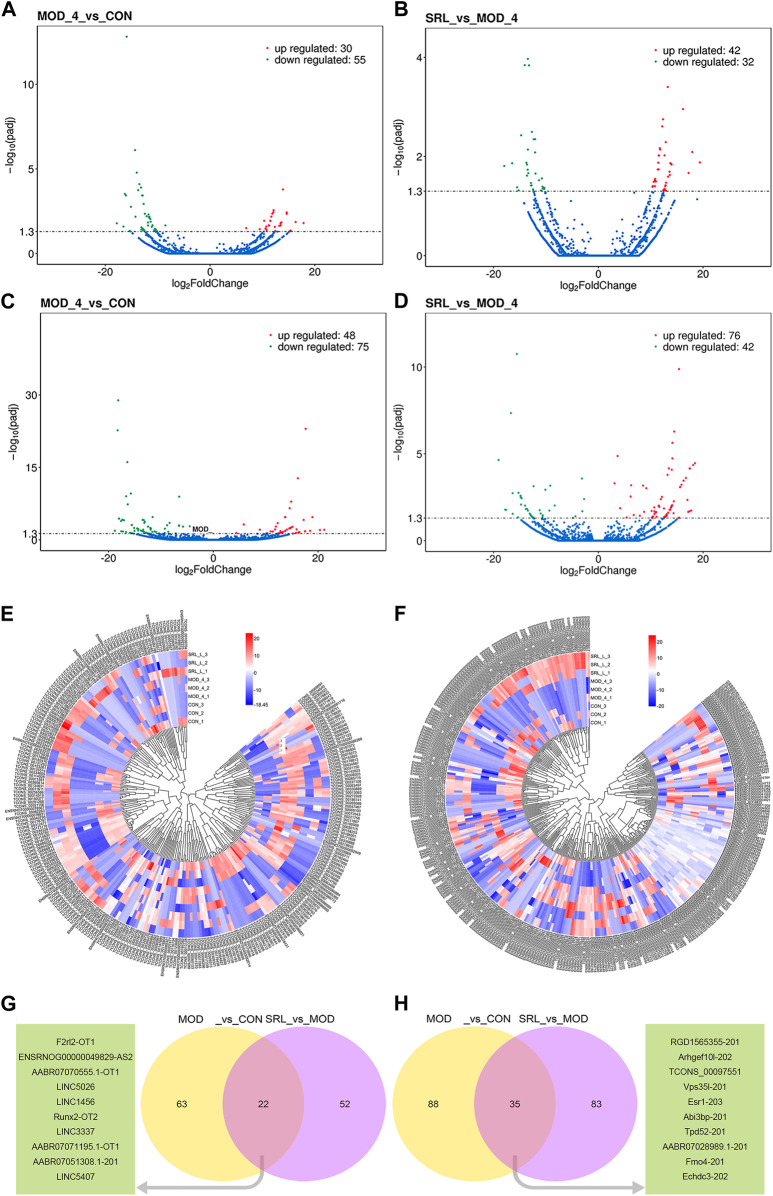
Analysis of differentially expressed genes. **(A–D)** Volcano plots of DElncRNA and DEmRNA in the MOD, CON, and SRL-4 groups. **(A)** DElncRNA between CON and MOD. **(B)** DElncRNA between MOD and SRL-4. **(C)** DEmRNA between CON and MOD. **(D)** DEmRNA between MOD and SRL-4. **(E,F)** Cluster analysis of lncRNA and mRNA differential expression profiles in the MOD, CON, and SRL-4 groups (heat map). Red: high expression; blue: low expression. **(E)** lncRNA. **(F)** mRNA. **(G,H)** Venn diagram of DElncRNAs and DEmRNAs, where the green portion represents the top 10 common genes (*p* < 0.05).

In this study, 85 DElncRNAs (Figure 4A) and 123 DEmRNAs (*p* <0.05; Figure 4C) were screened between the MOD and CON groups. Of these, 30 lncRNAs were upregulated and 55 lncRNAs were downregulated, whereas 48 mRNAs were upregulated and 75 mRNAs were downregulated. In addition, 74 DElncRNAs (Figure 4B), with 42 upregulated and 32 downregulated lncRNAs, and 118 DEmRNAs (Figure 4D), with 76 upregulated and 42 downregulated mRNAs, were identified by comparing the expression levels of MOD and SRL-4 (Supplementary File S1). Compared to the CON group, the differentially expressed RNAs in the SRL-4 group exhibit an increase in the number of upregulated RNAs and a decrease in the number of downregulated RNAs at the overall level. This indicates that changes have occurred in gene expression regulation under the treatment of SRL-4, which may involve specific biological processes and pathways. We performed clustering analysis on DElncRNAs (Figure 4E) and DEmRNAs (Figure 4F), and generated heat maps to visualize the results of the clustering analysis. The heatmap visually presents the global expression changes of multiple genes across multiple samples. Compared to MOD, the overall expression trend of both CON and SRL-4 is higher in terms of both DElncRNA and DEmRNA. This indicates that the overall gene expression trend of CON and SRL-4 is consistent when compared to MOD, and the expression profile of the drug-treated group tends towards that of the normal group. We plotted Venn diagrams of all DElncRNAs and DEmRNAs in the MOD, CON, and SRL-4 groups and identified 22 common DElncRNAs (Figure 4G) and 35 common DEmRNAs (Figure 4H) between the MOD and CON and MOD and SRL-4 groups, which may play more important regulatory roles in PF.

### 3.5 GO and KEGG enrichment analysis of DElncRNA and DEmRNA

**FIGURE 5:**
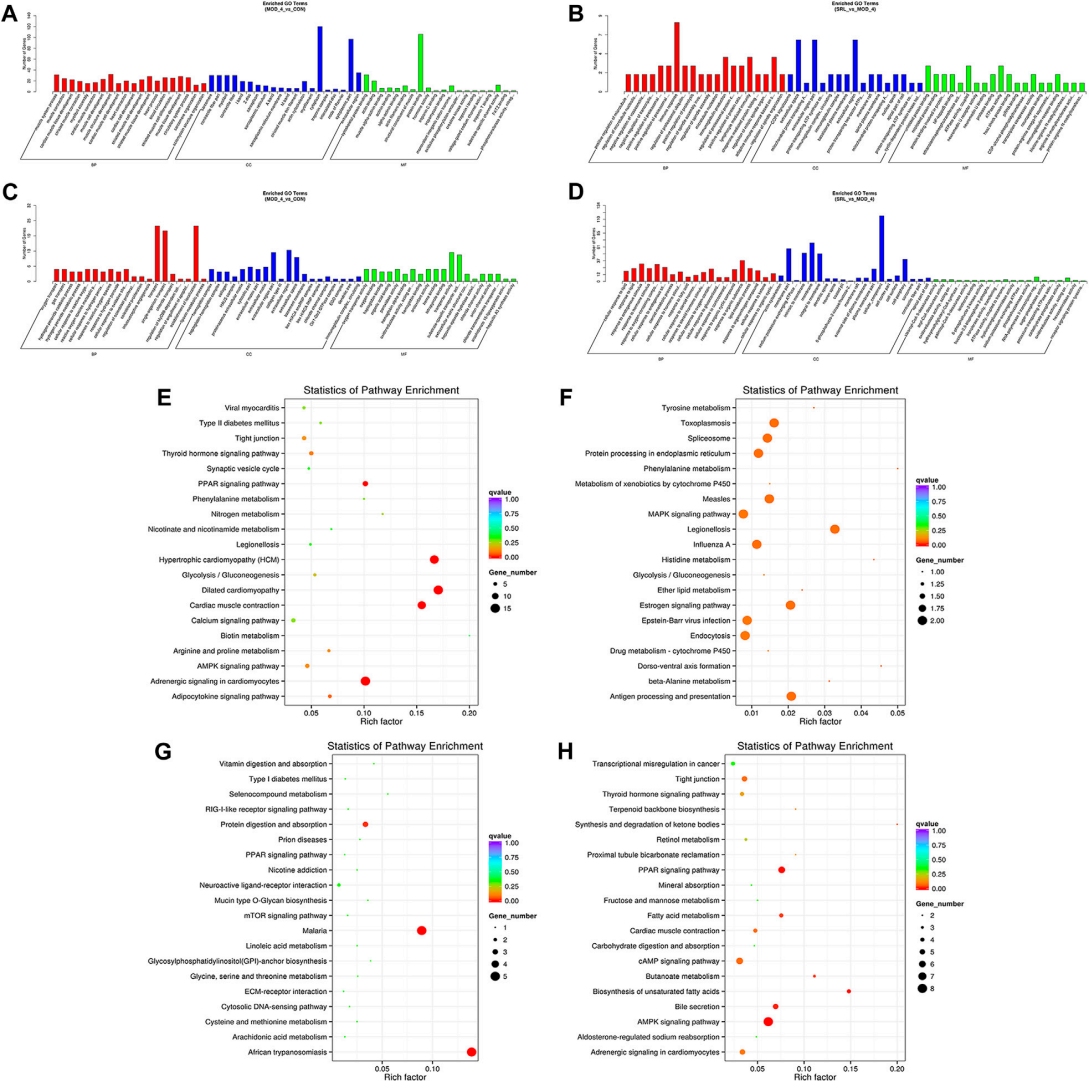
Functional enrichment analysis. **(A–D)** GO analysis of DElncRNA and DEmRNA in the MOD, CON, and SRL-4 groups. **(A)** DElncRNA in MOD and CON. **(B)** DElncRNA in MOD and SRL-4. **(C)** DEmRNA in MOD and CON. **(D)** DEmRNA in MOD and SRL-4. **(E–H)** KEGG analysis of DElncRNA and DEmRNA in the MOD, CON, and SRL-4 groups. **(E)** DElncRNA in MOD and CON. **(F)** DElncRNA in MOD and SRL-4. **(G)** DEmRNA in MOD and CON. **(H)** DEmRNA in MOD and SRL-4.

We performed functional enrichment analysis on DElncRNAs and DEmRNAs. GO enrichment analysis was performed on the co-located target genes of the DElncRNAs to predict their functions. The results showed that DElncRNAs were mainly enriched in the cytoplasm and protein-binding process in the MOD and CON groups (Figure 5A), whereas they were mainly enriched in the immune response, immunoglobulin production, lymphocyte-mediated immune process, and extracellular region in the MOD and SRL-4 groups (Figure 5B). GO enrichment analysis of DEmRNAs showed that the DEmRNAs in the MOD and CON groups were mainly enriched for transport, establishment of localization, extracellular region, transporter activity, and substrate-specific transporter activity (Figure 5C). The DEmRNAs between the MOD and SRL-4 groups were mainly enriched in cell membrane and organic substance responses (Figure 5D).

**FIGURE 6:**
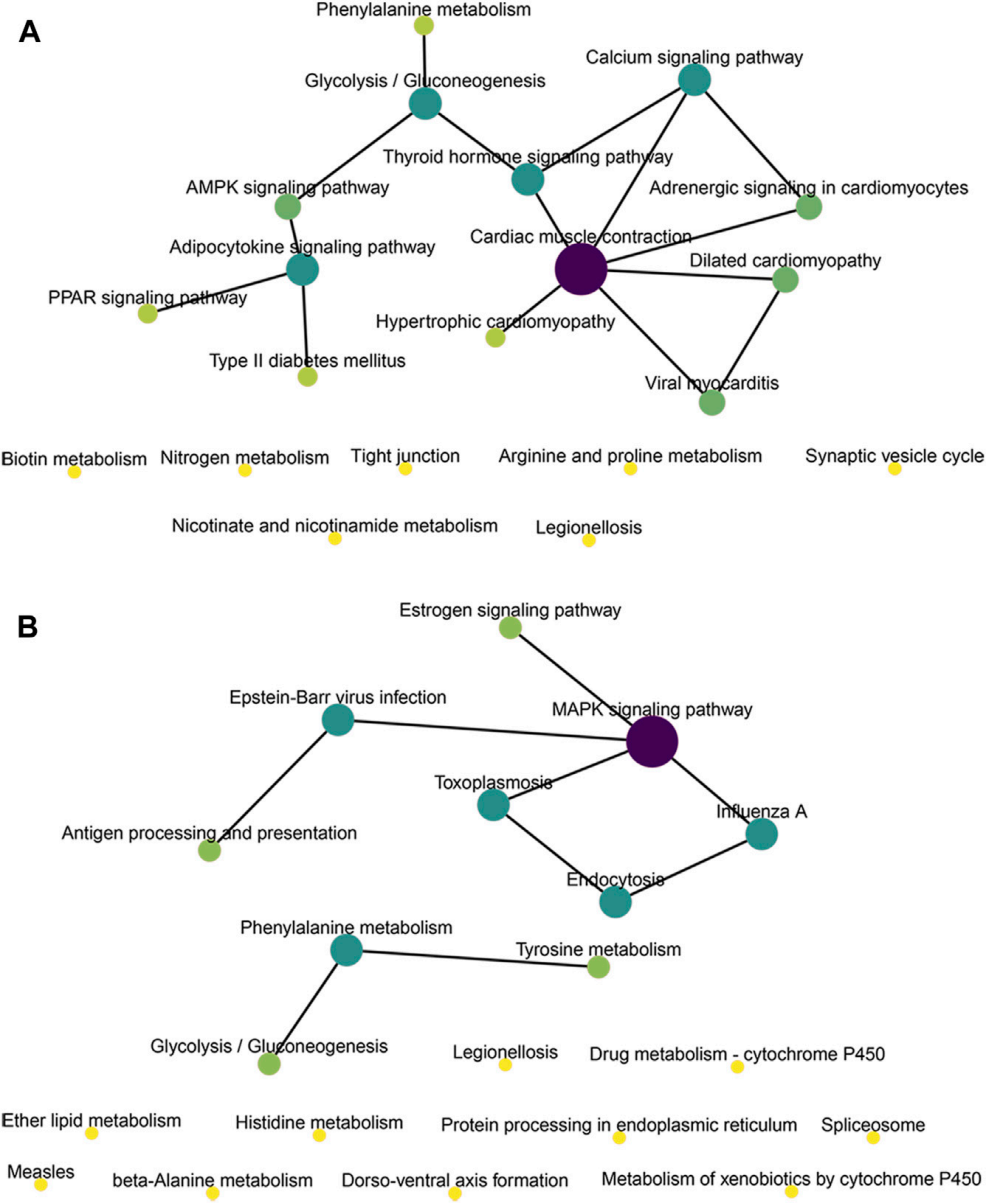
KEGG network diagram of target genes for co-location of DElncRNA in the MOD, CON, and SRL-4 groups. **(A)** MOD and CON. **(B)** MOD and SRL-4.

We conducted KEGG enrichment analysis on the co-located target genes of DElncRNAs and showed that DElncRNAs were mainly enriched in the adrenergic signaling pathway in hypertrophic cardiomyopathy (HCM), cardiac contraction, dilated cardiomyopathy, and myocardial cells in the MOD and CON groups (Figure 5E), whereas they were mainly enriched in legionellosis, antigen processing and presentation, and the MAPK signaling pathway in the MOD and SRL-4 groups. Additionally, influenza A, protein processing in the endoplasmic reticulum, antigen processing and presentation, and estrogen signaling pathways were significantly enriched (Figure 5F). In the KEGG analysis, DEmRNAs in the MOD and CON groups were mainly enriched in malaria, African trypanosomiasis, and protein digestion and absorption pathways (Figure 5G), whereas they were mainly enriched in the AMPK, PPAR, and cAMP signaling pathways in the MOD and SRL-4 groups. Most genes were enriched in the AMPK signaling pathway, indicating that it may play a crucial role in ameliorating the PF process (Figure 5H). Furthermore, we visualized the KEGG enrichment pathways in the MOD, CON, and SRL-4 groups and found that the cardiac contraction pathway had the highest connectivity in the CON and MOD groups (Figure 6A), whereas the MAPK signaling pathway had the highest connectivity in the MOD and SRL-4 groups (Figure 6B).

### 3.6 Construction of a lncRNA-miRNA-mRNA regulatory network

**FIGURE 7:**
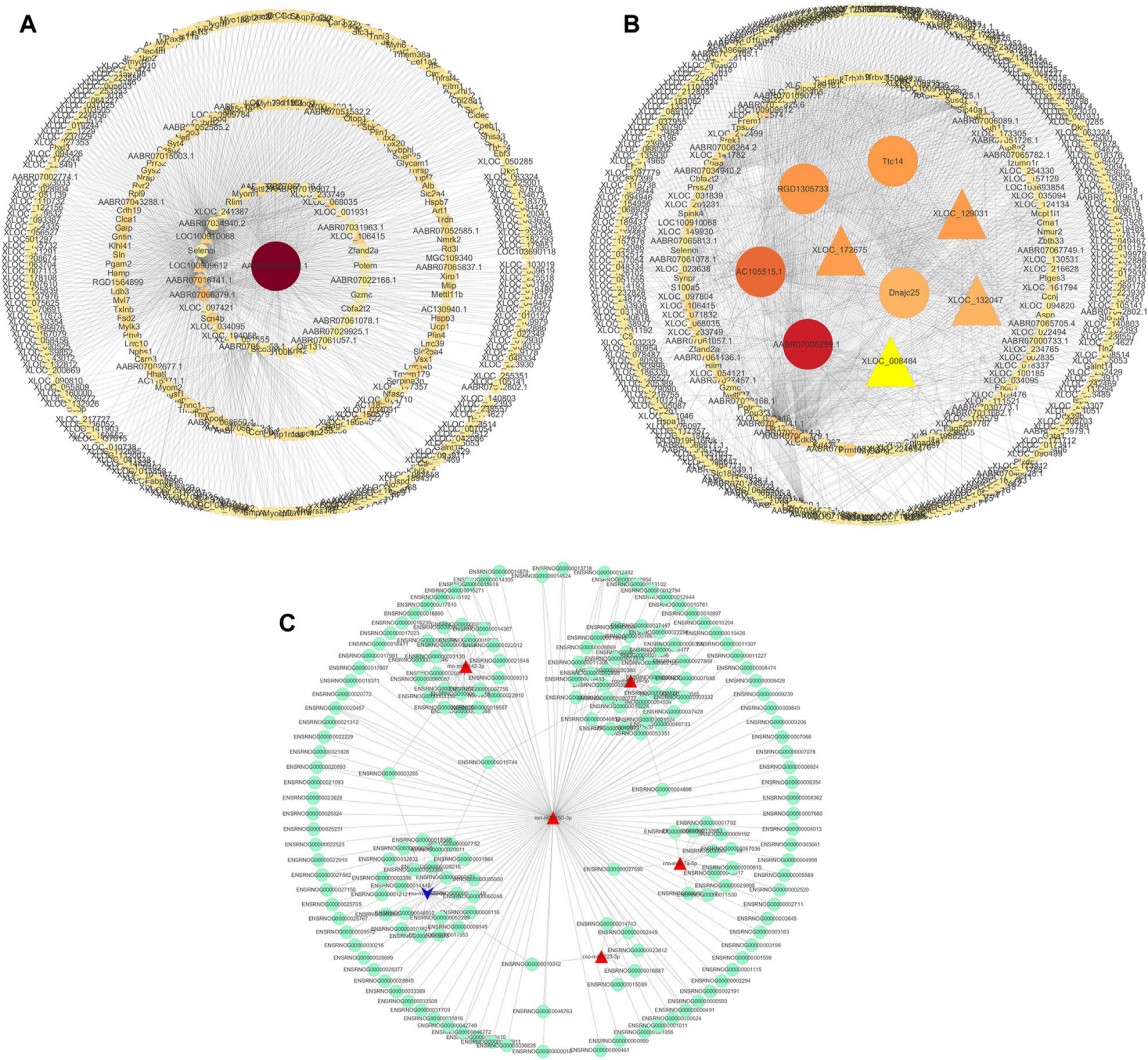
Visualization of gene regulatory networks. **(A,B)** DElncRNA and DEmRNA interaction network. Circles: lncRNA; triangles: mRNA. **(C)** miRNA-mRNA interaction network. Red: upregulation; blue: downregulation.

We used the Cytoscape software to construct a DElncRNA-DEmRNA interaction network (Figures 7A, B). Next, we combined miRNAs and mRNAs to construct a miRNA-mRNA interaction network, in which the top five miRNAs with the highest degree scores were ron-miR-150-3p, ron-miR-139-5p, ron-miR-433-3p, ron-miR-342-3p, and ron-miR-7a-5p (Figure 7C).

**FIGURE 8:**
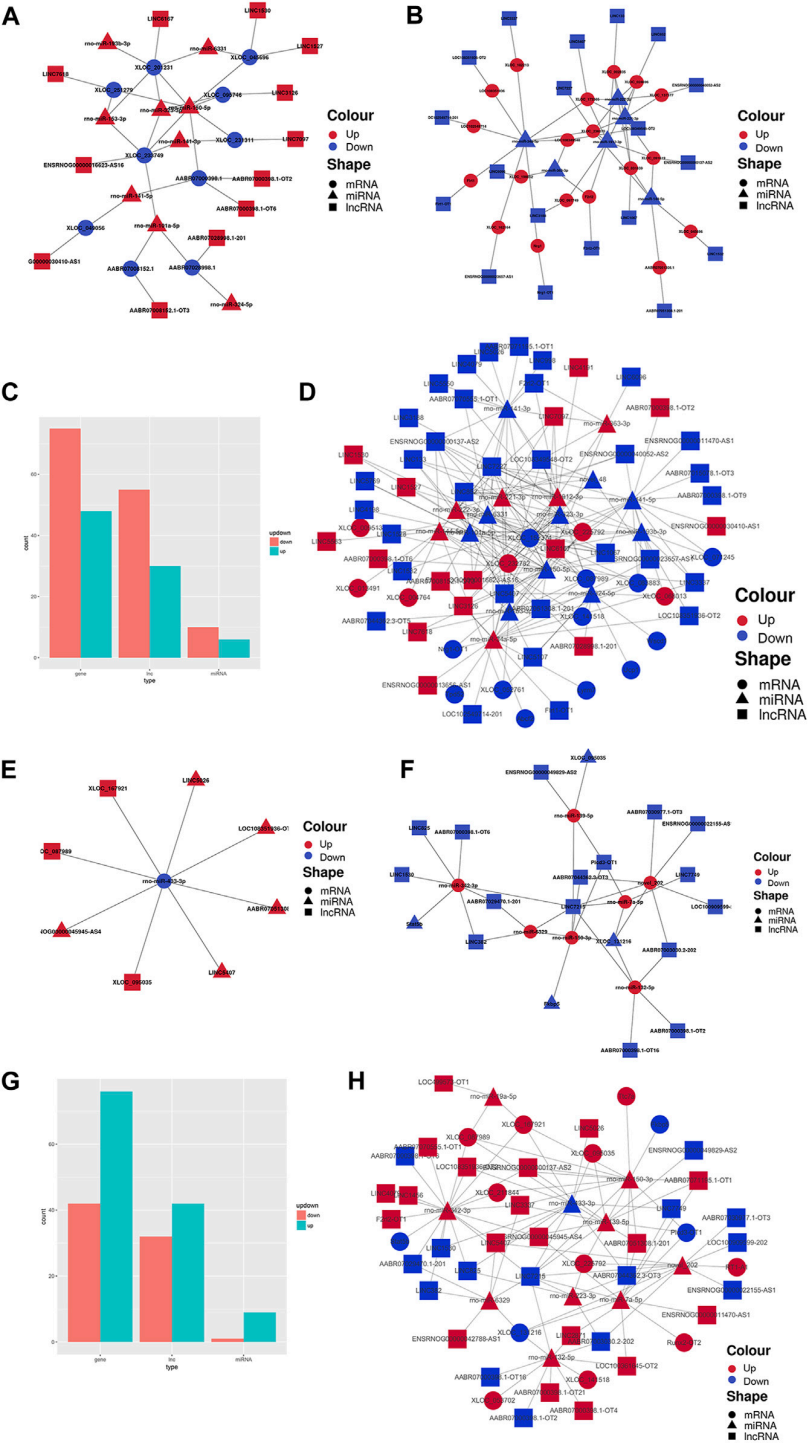
PF-related ceRNA network. **(A)** Upregulation of target lncRNA-downregulation of miRNA-upregulation of target genes between the CON and MOD groups. **(B)** Downregulation of target lncRNA-upregulation of miRNA-downregulation of target genes between the CON and MOD groups. **(C)** Summary histogram of the number of differentially expressed lncRNAs, miRNAs, and mRNAs differentially up- and downregulated between the CON and MOD groups. **(D)** Reciprocal network plot of target lncRNAs, miRNAs, and target genes expressed between the MOD and SRL-4 groups. **(E)** Upregulated target lncRNA-downregulated miRNA-upregulated target genes between the MOD and SRL-4 groups. **(F)** Downregulation of target lncRNA-upregulation of miRNA-downregulation of target genes between the MOD and SRL-4 groups. **(G)** Summary histogram of the number of differentially expressed lncRNAs, miRNAs, and mRNAs differentially up- and downregulated between the MOD and SRL-4 groups. **(H)** Reciprocal network diagram of differentially expressed target lncRNAs, miRNAs, and target genes between the MOD and SRL-4 groups.

Finally, to construct the entire ceRNA network, we analyzed differential information at the lncRNA level and obtained miRNA-related DElncRNAs and combined them with the miRNA-mRNA network to construct a lncRNA-miRNA-mRNA network (Figures 8A–E, H). The downregulation of the target Plcd3-OT1 and the upregulation of rno-miR-150-3p ultimately lead to the downregulation of the target Fkbp5 (Figure 8F). The upregulation of target AABR07051308.1-201 and F2rl2-OT1 affects the upregulation of rno-miR-342-3p, which ultimately leads to the downregulation of target Stat5b (Figure 8H). We then integrated the differential expression data of target lncRNAs, miRNAs, and target genes between different groups, and selected six key lncRNAs in the CON and MOD groups and seven key lncRNAs in the MOD and SRL-4 groups, based on the criteria of degree centrality (DC) and betweenness centrality (BC).

**FIGURE 9:**
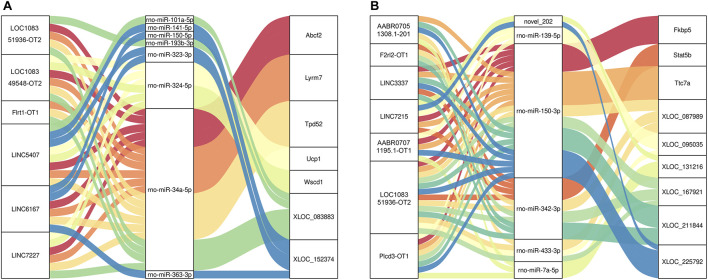
Sankey diagram of lncRNA-miRNA-mRNA regulatory relationships. **(A)** CON and MOD. **(B)** MOD and SRL-4.

The key lncRNAs in the CON and MOD groups were LOC108351936-OT2 (TCONS_00316378), LOC108349548-OT2 (TCONS_00005080), Flrt1-OT1 (TCONS_00034182), LINC5407 (TCONS_00229832), LINC6167 (TCONS_00265775), and LINC7227 (TCONS_00312477), while the key lncRNAs in the MOD and SRL-4 groups were AABR07051308.1-201 (ENSRNOT00000079208), F2rl2-OT1 (TCONS_00153261), LINC3337 (TCONS_00136842), LINC7215 (TCONS_00311807), AABR07071195.1-OT1 (TCONS_00303874), LOC108351936-OT2 (TCONS_00316378), and Plcd3-OT1 (TCONS_00054827). Based on these key lncRNAs, we plotted a Sankey diagram to visualize ceRNA network regulatory relationships (Figure 9).

## 4 Discussion

PF is a severe lung disease that causes irreversible lung damage, of which only 50% of patients survive for 2–3 years following diagnosis (Meyer, 2017). Medications and therapies can help alleviate symptoms and improve quality of life. Our previous research showed that SRL-4 can significantly improve pulmonary pathological changes in PF rats by delaying and reversing PF progression (Bai et al., 2021). In this study, we attempted to establish a lncRNA-miRNA-mRNA network with the biological functions of SRL-4 in PF using transcriptomic analysis. Our results will help to facilitate the development of PF treatment.

According to the ceRNA hypothesis, the lncRNA sequence is similar to that of the target miRNA and can regulate mRNA expression by acting as a miRNA sponge (Salmena et al., 2011). Previous studies have explored the role of ceRNAs in the prognosis and pathogenesis of fibrotic diseases. In a recent study, lncRNA-COX2 was shown to act as a ceRNA in EGR1. An increase in LncRNA-COX2 significantly reduces the expression of EGR1, displays anti-fibrotic effects, and has been identified as a therapeutic molecule for preventing abnormal epidural fibrosis (Yang et al., 2022). Importantly, overexpression of PFAL inhibited the expression of miR-18a, whereas silencing of PFAL had the opposite effect. PFAL promotes the activation and fibrosis of lung fibroblasts by acting as a ceRNA of miR-18a (Li et al., 2018). Huang et al. (2021) found that the knockdown of lncRNA Neat1 inhibited Cyth3 expression by regulating liver fibrosis and hepatic stellate cell activation through the ceRNA mechanism of miR-3a-148p and miR-3-22p. These results suggest lncRNAs as important regulatory factors in fibrosis. However, the potential molecular mechanisms leading to fibrosis remain largely unclear, and the regulatory networks are complex and require further research.

In the early stages of constructing the ceRNA network, we found that 74 lncRNAs, six miRNAs, and 118 mRNAs showed significant changes in expression in the MOD and SRL-4 groups. KEGG pathway enrichment analysis of DEmRNAs in the MOD and SRL-4 groups showed that these genes were mainly enriched in the AMPK, PPAR, and cAMP signaling pathways. A recent study showed that vaspin is a novel regulatory factor in hepatic steatosis that acts through the activation of AMPK via the GRP78 receptor, which effectively reduces hepatic fibrosis (Abdolahi et al., 2022). In addition, Cheng et al. (2021) confirmed that metformin reduced cell toxicity, inflammation, EMT, and fibroblast activation in PF by activating the AMPK signaling pathway, while Hua et al. (2021) suggested that icariin may have therapeutic potential for PF by inhibiting myofibroblast differentiation, possibly mediated by PPARγ. The cAMP/PKA pathway may act as a negative feedback regulator of ER stress-induced NLRP3 inflammasome activation, thereby inhibiting apoptosis in type II alveolar epithelial cells, ultimately alleviating PF (Hong et al., 2022). Therefore, we believe that the AMPK, PPAR, and cAMP signaling pathways have a strong influence on the pathogenesis of PF, providing new therapeutic targets for PF treatment.

MiRNAs are vital for regulating the epigenetic mechanisms of gene expression in PF. In the ceRNA network, we found that five miRNAs (ron-miR-150-3p, ron-miR-139-5p, ron-miR-433-3p, ron-miR-342-3p, and ron-miR-7a-5p) were significantly differentially expressed in the SRL-4 group and linked to the target mRNA. We also found that rno-miR-150-3p had high connectivity with the DE miRNAs of the ceRNA network. This finding suggests that rno-miR-150-3p is associated with the development of PF. Increased expression of serum miR-150-3p is associated with improved radiological lung damage in COVID-19 patients (Bueno et al., 2022) and overexpression of anti-tumor miR-150-3p in fibroblast-derived exosomes can inhibit the progression of liver cancer, suggesting that transferring miR-150-3p-loaded exosomes to liver cancer cells may become a new therapeutic option (Yugawa et al., 2021). Consistent with previous findings, our study showed that miR-150-3p was upregulated by 1.6-fold in PF and may affect the development of PF by directly affecting the expression of target genes. Similarly, other studies have explored the functions of five lncRNAs (ZFAS1, GAS5, LINC00941, MEG3, and ATB) in the occurrence and development of PF (Liu et al., 2020; Yang et al., 2020; Xu et al., 2021; Gao et al., 2022; Zhang et al., 2022) and found that their expression levels were highly correlated with the differentiation of PF fibroblasts and epithelial-mesenchymal transition.

In the ceRNA network, the DElncRNAs Plcd3-OT1 and LOC108351936-OT2 and DEmRNAs Fkbp5, Ttc7a and Stat5b showed high connectivity in the MOD and SRL-4 groups. We predicted that they may play important regulatory roles in PF. Plcd3 is a key enzyme in inositol phosphate metabolism and plays important roles in the cell cycle, proliferation, apoptosis, and cell movement (Xiang et al., 2019). Previous studies have shown that Plcd3 plays important roles in many biological processes such as the development of trophoblasts and cardiomyocytes, promotion of neurite outgrowth, and maintenance of normal cardiac function (Kouchi et al., 2011; Nakamura et al., 2014). In addition, Plcd3 has been shown to exert protective effects against oxidative damage in cardiomyocytes (Tappia et al., 2010; Nakamura et al., 2014). LOC108351936, also known as ribosomal protein L30-like, is the human ortholog of this gene in the gene library and is associated with herpes simplex. FK506 binding protein 5 (Fkbp5) is a stress-induced protein chaperone that affects biological processes through protein–protein interactions (Erlejman et al., 2014; Srivastava et al., 2015). The RNA expression of Ttc7a is relatively similar in many tissues, and studies have shown that patients with Ttc7a mutations have a phenotype specifically associated with intestinal and immune dysfunctions. STAT5b is a major regulator of the hematopoietic area and an essential signaling factor for the regulation and growth of hematopoietic extracellular tissue (Smith et al., 2022). However, currently, no reports exist on the relevance of these RNAs in fibrotic diseases; therefore, further analysis of the regulatory mechanisms of these genes is warranted.

MiRNAs and lncRNAs may be involved in the regulation of PF transcriptional processes in specific combinations. We found that Plcd3-OT1 competed with core rno-miR-150-3p to regulate the expression of target DEmRNAs in PF. Notably, our study identified an important lncRNA-miRNA-mRNA axis, the Plcd3-OT1/rno-miR-150-3p/Fkbp5 ceRNA axis. The expression levels and interactions in the PF were consistent with the ceRNA hypothesis. The target mRNA Fkbp5 regulated liver fibrosis induced by CCl4 in mice through mitochondrial autophagy (Zhang, 2017); Chen (2021) showed that FKBP5, as the host gene of lnc949, can regulate autophagy, while lnc949 can promote the expression of PF-related proteins and proliferation and migration, which can be reversed by FKBP5.

In addition, in the human sample data, Bueno et al. (2022) evaluated the serum expression of 754 miRNAs in 27 patients with COVID-19. At hospital discharge, 16 patients had radiological pulmonary improvement. They observed that increased expression of miR-150 was associated with pulmonary improvement at hospital discharge. Furthermore, Zang et al. (2023) utilized peripheral blood samples from idiopathic pulmonary fibrosis (IPF) patients to identify non-coding RNAs and mRNAs. They discovered that miR-150-5p, as a critical miRNA in the ceRNA network, was closely linked to lung disease or fibrosis and might play a significant role in IPF. These findings were aligned with our results and conclusions. Moreover, Zhang et al. (2020) revealed that the expression of lncRNA NEAT1 was negative correlated with the expression of miR-9-5p in human PF tissues and TGF-β1-induced cells. NEAT1 could also directly target miR-9-5p to regulate TGF-β1-induced PF. Sun et al. (2019) discovered that the lncRNA PFAR acted as a sponge for miR-15a to execute the function of ceRNA and promote fibrosis by lung fibroblasts. This study unveiled a potential regulatory network involving the cRNA PFAR and miR-15a. Additionally, Lactotransferrin (LTF) was found to be significantly upregulated in radioresistant squamous cell lung carcinoma clinical samples and cell lines. LTF can directly interact with AMPK to facilitate its phosphorylation and activate autophagy signaling. This suggests that LTF might serve as a target for radiotherapy sensitization in lung cancer (Wen et al., 2023). These human data indirectly corroborate our findings. However, it will be necessary to further validate our other findings using more human samples.

## 5 Conclusion

By integrating various differentially expressed datasets, herein, we successfully constructed a ceRNA network comprising 35 lncRNAs, 10 miRNAs, and 13 mRNAs. The lncRNA-miRNA-mRNA network has the potential to serve as a biomarker and therapeutic target for improving PF in patients with SRL-4. Our results also suggest that AMPK, PPAR, and cAMP signaling pathways are important for improving PF through SRL-4. Further investigations are warranted to validate our findings.

## Data Availability

The original contributions presented in the study are included in the article/Supplementary Material; further inquiries can be directed to the corresponding author.
